# Developing a Preliminary Clinical Prediction Model for Prognosis of Pneumonia Complicated with Heart Failure Based on Metagenomic Sequencing

**DOI:** 10.1155/2023/5930742

**Published:** 2023-07-18

**Authors:** Rongyuan Yang, Yong Duan, Dawei Wang, Qing Liu

**Affiliations:** ^1^The Second Clinical School of Medicine, Guangzhou University of Chinese Medicine, Guangdong Provincial Hospital of Chinese Medicine-Zhuhai Hospital, Guangzhou, Guangdong 519015, China; ^2^Department of Cardiovascular Medicine, The Third Hospital of Changsha, Changsha 410000, China; ^3^The First Affiliated Hospital of Guangzhou University of Chinese Medicine, Guangzhou, Guangdong 510405, China

## Abstract

**Background:**

The predictive factors of prognosis in patients with pneumonia complicated with heart failure (HF) have not been fully investigated yet, especially with the use of next-generation sequencing (NGS) of metagenome.

**Methods:**

Patients diagnosed with pneumonia complicated with HF were collected and divided into control group and NGS group. Univariate and multivariate logistic regression and LASSO regression analysis were conducted to screen the predictive factors for the prognosis, followed by nomogram construction, ROC curve plot, and internal validation. Data analysis was conducted in SPSS and R software.

**Results:**

The NGS of metagenome detected more microbial species. Univariate and multivariate logistic regression and LASSO regression analysis revealed that Enterococcus (*χ*^2^ = 7.449, *P* = 0.006), Hb (Wals = 6.289, *P* = 0.012), and ProBNP (Wals = 4.037, *P* = 0.045) were screened out as potential predictive factors for the prognosis. Nomogram was constructed with these 3 parameters, and the performance of nomogram was checked in ROC curves (AUC = 0.772). The specificity and sensitivity of this model were calculated as 0.579 and 0.851, respectively, with the threshold of 0.630 in ROC curve. Further internal verification indicated that the predictive value of our constructed model was efficient.

**Conclusion:**

This study developed a preliminary clinical prediction model for the prognosis of pneumonia complicated with HF based on NGS of metagenome. More objects will be collected and tested to improve the predictive model in the near future.

## 1. Introduction

As the population ages, community-acquired pneumonia affects more than 5 million and up to 100,000 deaths annually in the USA [1]. Evidence suggests that pneumonia is associated with long-term cardiovascular outcome, especially heart failure (HF) [1–3]. Investigation on the clinical trials of PARADIGM-HF [4] and PARAGON-HF [5] demonstrated that pneumonia incidence was high in patients with HF followed by 4-fold higher mortality [6]. However, the predictive factors of prognosis in patients with pneumonia complicated with HF were not fully investigated yet [7–10], especially with the help of next-generation sequencing (NGS) of metagenome.

This study collected patients with pneumonia complicated with HF, and tried to screen the predictive factors for the clinical prognosis of HF with pneumonia [11–13], based on the next-generation sequencing (NGS) of metagenome to detect microbial pathogens. Then, the univariate and multivariate logistic regression, least absolute shrinkage selector operator (LASSO) regression analysis, nomogram, receiver operating characteristic (ROC) curve plot, and internal validation were performed to construct the visualization model and validation. Thus, we aimed to develop a preliminary clinical prediction model for the prognosis of pneumonia complicated with HF based on NGS of metagenome and hope that this model will be helpful to the assessment of prognosis of HF with pneumonia [8].

## 2. Methods

### 2.1. Patients Selection and Data Collection

Initially, 66 patients diagnosed with pneumonia complicated with HF in Guangdong Hospital of Traditional Chinese Medicine from January 2021 to October 2022 were collected and divided into two groups (33 cases each), i.e., sputum culture test group (control group) and sputum culture combined with NGS test of metagenome group (NGS group). The samples for the sputum culture were obtained from nasopharynx or oropharynx, while the samples for the NGS were obtained from fibre bronchoscopy examination.

### 2.2. Ethical Approval

This study was approved by the Ethics Committee of Guangdong Provincial Hospital of Traditional Chinese Medicine with the approval registration number BF2022-115.

### 2.3. Inclusion and Exclusion Criteria

Patients were collected and divided into the group according to the diagnostic, inclusion, and exclusion criteria. The diagnosis of pneumonia refers to the Guidelines for the Diagnosis and Treatment of Community acquired Pneumonia in China (Supplementary Table 2), and the diagnosis of HF refers to the Guidelines for the Diagnosis and Treatment of HF in China.

Inclusion criteria: (1) The condition conforms to the diagnostic criteria. (2) The cardiac function is classified as II-IV according to NYHA. (3) The patient voluntarily participated and signed the relevant consent form. Exclusion criteria: (1) Patients with abnormal mental consciousness who cannot cooperate or patients with unstable vital signs. (2) Patients with related drug contraindications or allergies. (3) Those who participate in other clinical trials within 1 month. (4) Pregnant or ready to be pregnant, lactating women, or infants.

### 2.4. Univariate and Multivariate Logistic Regression Analysis

Regarding the prognosis (i.e., alive and dead) of patients, binary logistic regression with univariate and multivariate analysis was conducted to check the predictive factors, including demographic information, complications, microbial species, and biochemistry parameters. The variables with *P* < 0.1 in univariate analysis were screened out for multivariate analysis and *P* < 0.05 in multivariate analysis were taken as potential prognostic predictors.

### 2.5. Construction and Internal Verification of Nomogram

The variables with *P* < 0.05 in multivariate logistic regression were screened out for the preparation of constructing nomogram. Meanwhile, the LASSO regression analysis was performed to select the best predictive model with the least variables by calculating the values of *λ*.min and *λ*.1se. Then, the ROC curves were constructed to evaluate the predictive performance of the nomogram, with the calculated area under curves (AUC) of ROC indicating the model performance. Internal verification of the nomogram was tested with the whole dataset, and predictive value for the prognosis (alive or dead) was clustered to 2 main categories by the median method.

### 2.6. Statistical Analysis

Data were analyzed in SPSS (v26.0, Inc., Chicago, Illinois, USA) and R (v3.6.2, http://www.r-project.org) software. The continuous data were expressed as mean ± standard deviation, and the comparison between the two groups was performed by two independent samples Student's *t*-test. The categorical variables were expressed in frequency and proportions (%), and Chi-square tests were performed for comparison between the groups. Univariate and multivariate logistic regression analysis was conducted according to binary regression analysis in SPSS. The forest plot, violet plot, LASSO regression, nomogram, and ROC curves were plotted in the R software. *P* < 0.05 was considered as statistical significance.

## 3. Results

### 3.1. Demographic Characteristics of the Selected Patients

**Figure 1:**
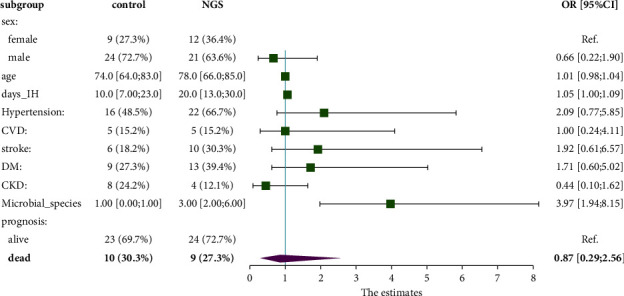
Comparison and forest plot of the baseline characteristics of the patients.

Totally, 66 patients were enrolled based on the criteria. For the parameters of demographic characteristics, there was no significant difference between the control group (*n* = 33) and NGS group (*n* = 33), except the days in the hospital (*P* = 0.033) **(**Figure 1 and Table 1**)**.

### 3.2. Microbial Species Detected by NGS

**Figure 2:**
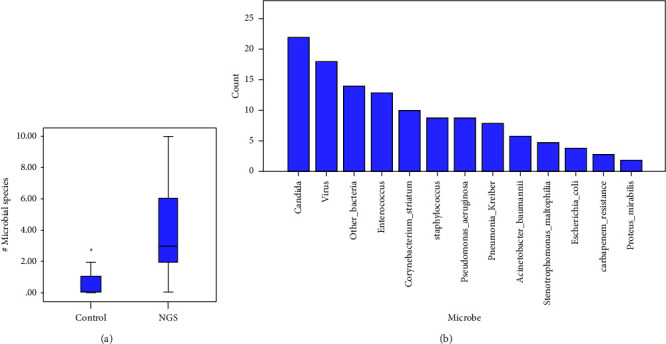
The detected microbial species in the patients with pneumonia complicated with HF. (a) Comparison of the detected microbial species by the NGS versus control groups. (b) Column plot for the count of each microbe detected in all the patients.

Compared with the sputum culture group, the NGS group detected more microbial species (Supplementary Table 1 and Figure 2(a)**)**. The 3 most commonly detected bacteria were *Candida*, Enterococcus, and *Corynebacterium striatum* (Figure 2(b)).

### 3.3. Univariate and Multivariate Logistic Regression of Parameters on Clinical Treatment and Prognosis

Firstly, logistic regression was used to analyze the influence of pathogen type on the clinical treatment effects such as antibiotics. Results of the univariate logistic regression showed that Enterococcus may significantly affect the clinical treatment effects (*χ*^2^ = 9.48, *P* = 0.009), while this significance disappeared in the multivariable logistic regression (*χ*^2^ = 0.998, *P* = 0.32) (Table 2). Then, logistic regression was also performed to analyze the influence of pathogen type on clinical prognosis (i.e., alive or dead). Both univariate and multivariable logistic regression showed that Enterococcus may significantly affect the clinical prognosis (*χ*^2^ = 7.449, *P* = 0.006); thus, Enterococcus was selected as one of the factors affecting clinical prognosis (Table 3).

**Figure 3:**
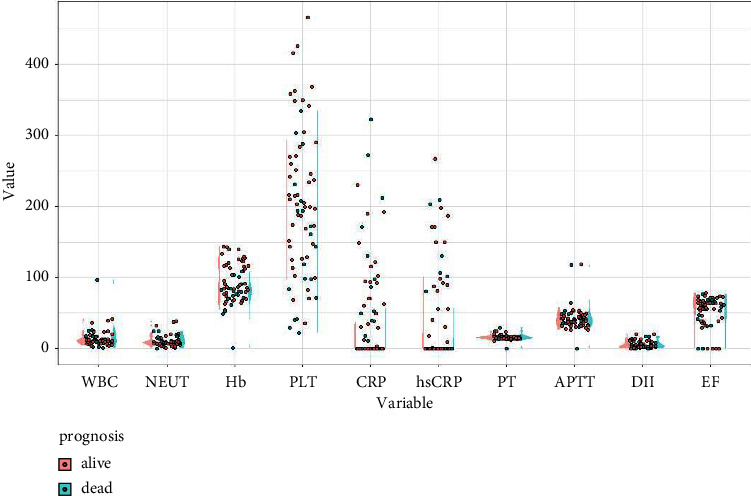
Comparison of the biochemical parameters in the patients with prognosis of alive versus dead.

Secondly, the levels of biochemical parameters in patients with different prognosis are visualized in Figure 3. Then, the influence of biochemical parameters on clinical prognosis was examined. Univariate regression analysis initially identified hemoglobin (Hb) (*P* = 0.011), platelet (PLT) (*P* = 0.013), C-reactive protein (CRP) (*P* = 0.062), prohormone of brain natriuretic peptide (proBNP) (*P* = 0.052), alanine aminotransferase (ALT) (*P* = 0.067), and Aspartate transferase (AST) (*P* = 0.081) as biochemically prognostic factors for the clinical prognosis (Table 4). Then, multivariable logistic regression screened out Hb (Wals = 6.289, *P* = 0.012) and ProBNP (Wals = 4.037, *P* = 0.045) as biochemically prognostic factors (Table 5).

### 3.4. Construction and Visualization by Nomogram for Multivariate Logistic Regression

**Figure 4:**
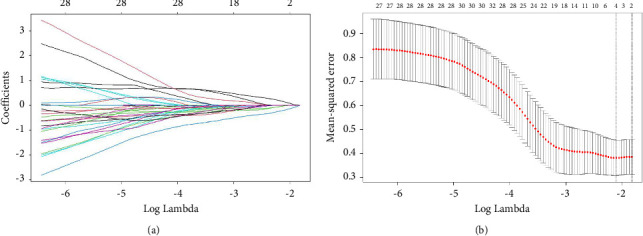
The LASSO regression (a) and cross-validation (b) for parameters selected in the LASSO model.

The LASSO regression analysis was used to find the most appropriate model with the least parameters. As the coefficient value of screened variables decreased to zero when *λ* increased, the contributions of most variables could be eliminated in this model **(**Figure 4(a)**)**. Then, cross-validation was conducted to choose the best performance model composed with least variables, and the partial-likelihood deviance curve showed that 3 variables were enrolled for the best model (Figure 4(b)).

**Figure 5:**
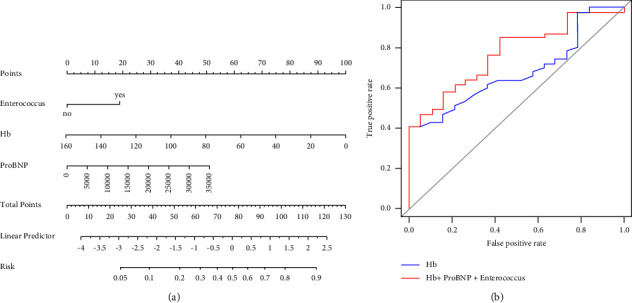
Construction of the clinical prediction model with selected parameters and efficacy test. (a) The nomogram was constructed using multivariate regression variables. (b) Receiver operating characteristic (ROC) curve of the nomogram.

Meanwhile, regarding to the results of multivariate logistic regression on clinical treatment and prognosis abovementioned, the 3 parameters of Enterococcus, Hb, and ProBNP were finally selected to construct the nomogram for visualization (Figure 5(a)). To check the predictive efficacy of this model, the ROC curves of Hb independently and the combination of Hb, Enterococcus, and ProBNP were both plotted. As we can see, the AUC of the 3 parameters (AUC = 0.772) prediction was better than that of the Hb independently (AUC = 0.677) under the ROC curves (Figure 5(b)). The specificity and sensitivity of this 3 parameters model were calculated as 0.579 and 0.851, respectively, with the threshold of 0.630 in ROC curve.

### 3.5. Internal Verification of Nomogram Prediction

**Figure 6:**
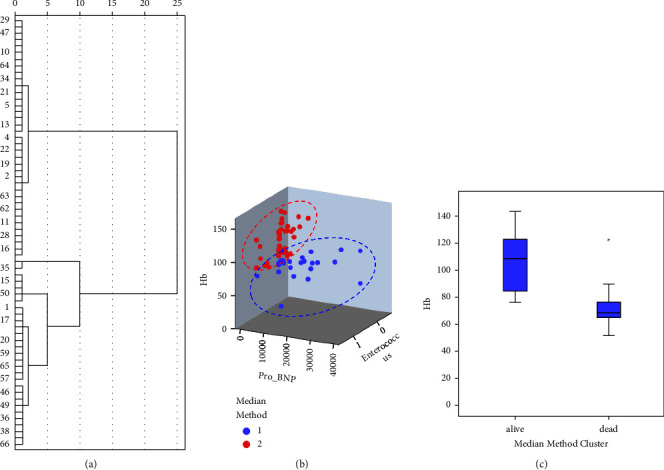
Classification of patients by the predictive value of multivariate regression in the clinical prediction model. (a) Dendrogram to cluster patients by median method based on model prediction. (b) The 3D scatter plot of patients by median method clustering based on model prediction. (c) Comparison of Hb in the groups clustered by median method based on model prediction.

The whole dataset was used for the internal verification of the nomogram prediction. The predictive value was calculated for the prognosis of alive or dead, and the result was clustered by the median method, exhibiting 2 main categories clustered in the dendrogram (Figure 6(a)). Then, the 3 parameters of Hb, Enterococcus, and ProBNP were clustered and shown in the 3D scatter plot, and we can find that the 2 main categories were separated quite independently (Figure 6(b)). When checking Hb independently, the levels of Hb in the 2 categories were significantly different (Figure 6(c)). These data of internal verification indicated that the predictive value of our constructed model was efficient.

## 4. Discussion

This study tried to develop a preliminary clinical prediction model for the prognosis of pneumonia complicated with HF based on NGS of metagenome. As we expected, the NGS group detected more microbial species. Univariate and multivariate logistic regression and LASSO regression analysis revealed that 3 parameters, i.e., Enterococcus, Hb, and ProBNP were screened out as potential predictive factors for the prognosis. The nomogram was constructed with these 3 parameters, and the performance of the nomogram was checked in the ROC curves and validated by internal verification.

Heart failure is a broad catch of all terms and various conditions and etiologies which can lead to poor pump function and low perfusion. The diagnosis and etiology of heart failure could easily be obtained from the chart review of the patients. There are several factors for monitoring and prognosis of HF with pneumonia [14–16], in which ProBNP is a classic factor [17]. Meanwhile, Hb is reported to be an independent predictor for the survival in patients with chronic HF (CHF), with anaemic and polycythaemic patients having the worst survival in the ELITE II trial [18]. The clinical trial named EMPEROR-Reduced also evidenced that anemia was associated with poor outcomes of HF, and empagliflozin administration showed improved HF and kidney outcomes irrespective of anemia status at baseline [19]. A similar result was observed on the HF patients with iron deficiency or abnormal red cell indices [20]. Besides, Hb is reported to be associated with the frailty score in community-acquired pneumonia, which may affect the prognosis of pneumonia [21]. Our data are consistent with these reports that the low level of Hb was associated with poor outcomes of HF.

Bacteria are recognized as one of the predictive factors for the prognosis of HF. Bacterial infection can indirectly cause HF by inducing endocarditis, myocarditis, and infections in other organs including pneumonia. In our study, myocarditis may explain the cause of heart failure in one aspect. Endocarditis could be diagnosed with the suspicion of bacteremia and echocardiographic changes. The bacteria can also lodge on heart valves and cause infection of the endocardium [22, 23]. It is demonstrated that procalcitonin (PCT)-based indication of bacterial infection identifies high risk acute HF (AHF) patients, and elevated PCT indicated probable bacterial infection with poorer in-hospital and postdischarge outcomes, despite similar severity of HF [24].

Metagenomic next-generation sequencing has been widely used for pathogen determination from patients with infectious diseases, especially pneumonia, and identification of specific pathogens can guide the antimicrobial treatments [25]. Our results showed that NGS detected more kinds of microbe species compared with the normal sputum culture, and univariate and multivariate logistic regression analysis revealed that the infection of Enterococcus detected by the NGS was statistically significant related to the clinical outcome. Besides, Enterococcus infection is reported to be associated with HF in the literature. Although the major source of Enterococcal endocarditis is from genitourinary tract infections which are more common than pneumonia, it is reported that Enterococcal endocarditis was one of the subacute infection characterized by HF [26]. It is also recommended to treat pneumonia similar to Enterococcal infection in the patients with HF and Enterococcal infection in other organs with HF [27]. Therefore, the reports support our data which suggest that bacteria are associated with the prognosis of HF, and untreated Enterococcus infection in pneumonia may become a predictor for poor prognosis of patients with HF in the hospital.

In conclusion, our study developed a preliminary clinical prediction model and used visualized nomogram for the prognosis of pneumonia complicated with HF based on NGS of metagenome. However, the main limitation of this study is the relatively small sample size. In the future projects, more objects will be collected and tested to improve the predictive model and internal validation and consummate the external validation.

## Figures and Tables

**Table 1:** Comparison of the baseline characteristics of the patients.

	Control	NGS	OR	*P* value
	*n* = 33	*n* = 33		
Sex				0.597
Female	9 (27.3%)	12 (36.4%)	Ref.	
Male	24 (72.7%)	21 (63.6%)	0.66 [0.22; 1.90]	
Age	74.0 [64.0; 83.0]	78.0 [66.0; 85.0]	1.01 [0.98; 1.04]	0.281
Days in the hospital	10.0 [7.00; 23.0]	20.0 [13.0; 30.0]	1.05 [1.00; 1.09]	0.033^*∗*^
Hypertension	16 (48.5%)	22 (66.7%)	2.09 [0.77; 5.85]	0.213
CVD	5 (15.2%)	5 (15.2%)	1.00 [0.24; 4.11]	1
Stroke	6 (18.2%)	10 (30.3%)	1.92 [0.61; 6.57]	0.389
DM	9 (27.3%)	13 (39.4%)	1.71 [0.60; 5.02]	0.433
CKD	8 (24.2%)	4 (12.1%)	0.44 [0.10; 1.62]	0.338
Comorbidities	3.00 [2.00; 4.00]	3.00 [1.00; 3.00]	0.91 [0.63; 1.33]	0.566
WBC	11.6 (7.37)	16.6 (16.6)	1.04 [0.98; 1.11]	0.125
NEUT	9.36 (6.71)	11.9 (8.45)	1.05 [0.98; 1.12]	0.176
Hb	92.9 (25.9)	87.1 (28.5)	0.99 [0.97; 1.01]	0.393
PLT	213 (112)	190 (96.2)	1.00 [0.99; 1.00]	0.386
CRP	115 (103)	90.1 (59.6)	1.00 [0.99; 1.00]	0.445
hsCRP	91.4 (60.9)	142 (87.1)	1.01 [1.00; 1.02]	0.167
ProBNP	6792 (9136)	6244 (6423)	1.00 [1.00; 1.00]	0.788
PT	15.4 (2.12)	15.8 (3.60)	1.05 [0.88; 1.24]	0.609
APTT	45.4 (20.6)	39.3 (7.40)	0.97 [0.92; 1.01]	0.118
DII	5.39 (4.70)	5.64 (5.53)	1.01 [0.91; 1.12]	0.851
ALT	197 (825)	43.0 (49.7)	1.00 [0.99; 1.00]	0.299
AST	301 (1439)	66.6 (84.3)	1.00 [1.00; 1.00]	0.364
Cr	187 (225)	179 (131)	1.00 [1.00; 1.00]	0.864
ST_T	17 (51.5%)	10 (30.3%)	0.42 [0.15; 1.14]	0.133
EF	61.0 [41.0; 69.0]	63.0 [55.5; 66.5]	1.03 [0.98; 1.07]	0.574
CPIS_L				0.082
Score0	1 (3.03%)	1 (3.12%)	Ref.	
Score1	8 (24.2%)	2 (6.25%)	0.29 [0.01; 14.5]	
Score2	24 (72.7%)	29 (90.6%)	1.20 [0.03; 48.7]	
Efficacy				0.136
Effective	2 (6.06%)	1 (3.03%)	Ref.	
Valid	19 (57.6%)	12 (36.4%)	1.19 [0.09; 40.2]	
Invalid	12 (36.4%)	20 (60.6%)	3.05 [0.22; 103]	
Prognosis				1
Alive	23 (69.7%)	24 (72.7%)	Ref.	
Dead	10 (30.3%)	9 (27.3%)	0.87 [0.29; 2.56]	

*Note.* ^*∗*^Comparison between the two groups, *P* < 0.05.

**Table 2:** Regression analysis of the influence of pathogen type on the clinical treatment effect.

Pathogens	Univariate analysis			Multivariate analysis		
	−2 log likelihood	*χ* ^2^	*P* value	−2 log likelihood	*χ* ^2^	*P* value
*Candida*	13.744	1.568	0.457	43.137	0.998	0.607
Enterococcus	19.211	9.48	0.009^*∗*^	44.420	2.281	0.32
*Corynebacterium striatum*	12.434	0.819	0.664	44.229	2.09	0.352
*Staphylococcus*	12.326	0.803	0.669	43.864	1.724	0.422
*Pseudomonas aeruginosa*	11.078	0.997	0.607	42.635	0.496	0.78
*Pneumonia kreiber*	12.562	1.179	0.555	45.204	3.065	0.216
Microbial species	40.308	18.181	0.444	56.028	13.889	0.736
Virus	12.41	0.345	0.841	42.813	0.674	0.714

*Note.* ^*∗*^Multiple logistic regression analysis, *P* < 0.05.

**Table 3:** Regression analysis of the influence of pathogen type on clinical prognosis.

Pathogens	Univariate analysis		Multivariate analysis	
	Value	*P* value	Value	*P* value
*Candida*	0.148	0.701	0.283	0.594
Enterococcus	7.449	0.006^*∗*^	7.449	0.006∗
*Corynebacterium striatum*	2.029	0.154	3.42	0.064
*Staphylococcus*	0.219	0.64	0.057	0.811
*Pseudomonas aeruginosa*	0.105	0.746	0.072	0.789
*Pneumonia kreiber*	0.337	0.562	0.01	0.919
Microbial species	0.015	0.902	1.485	0.223
Virus	0.451	0.502	0.698	0.404

*Note.* ^*∗*^Binary logistic regression analysis, *P* < 0.05.

**Table 4:** Univariate regression analysis of the influence of biochemical parameters on clinical prognosis.

Parameters	Value	*P* value
WBC	1.567	0.211
NEUT	0.003	0.955
Hb	6.519	0.011^*∗*^
PLT	6.236	0.013^*∗*^
CRP	3.495	0.062^*∗*^
hsCRP	1.529	0.216
ProBNP	3.772	0.052^*∗*^
PT	0.056	0.813
APTT	0.175	0.676
DII	0.011	0.917
ALT	3.365	0.067^*∗*^
AST	3.05	0.081^*∗*^
Cr	2.393	0.122
ST_T	0.461	0.497
EF%	1.118	0.29
CPIS_L	0.789	0.674

*Note.* ^*∗*^Single-factor analysis of binary logistic regression, *P* < 0.1.

**Table 5:** Multivariate regression analysis of the influence of biochemical parameters on clinical prognosis.

	B	S. E	Wals	*P* value	Exp (*B*)	Exp (*B*) 95% C.I.	
						Lower limit	Upper limit
Hb	0.04	0.016	6.289	0.012^*∗*^	1.041	1.009	1.074
ProBNP	0	0	4.037	0.045^*∗*^	1	1	1
Constant	−2.192	1.331	2.711	0.1	0.112		

*Note.* ^*∗*^Multiple factor analysis of binary logistic regression, *P* < 0.05.

## Data Availability

All the data used in this study are available from the corresponding author upon reasonable request.

## Supplementary Materials

Supplementary Materials Supplementary Table 1: Distribution of pathogens detected by the sputum culture or NGS. Supplementary Table 2: The diagnostic criteria for pneumonia diagnosis in the study.
