# Supplementary material for: Developing a Preliminary Clinical Prediction Model for Prognosis of Pneumonia Complicated with Heart Failure Based on Metagenomic Sequencing
Source: Crit Care Res Pract. 2023 Jul 18;2023:5930742. doi: 10.1155/2023/5930742 (PMC10368513; doi:10.1155/2023/5930742)
Supplement: Supplementary Materials — Supplementary Table 1: Distribution of pathogens detected by the sputum culture or NGS. Supplementary Table 2: The diagnostic criteria for pneumonia diagnosis in the study. [file 5930742.f1.docx]

**Supplementary Table 1 Distribution of pathogens detected by sputum culture or NGS.**

| **Sputum culture** | | | |  | **NGS** | | | |
| --- | --- | --- | --- | --- | --- | --- | --- | --- |
| **Pathogen*** | **Frequency** | **Effective%** | **Cumulative%** |  | **Pathogen** | **Frequency** | **Effective%** | **Cumulative%** |
| 0 | 16 | 48.5 | 48.5 |  | 0 | 1 | 3.0 | 3.0 |
| 1 | 12 | 36.4 | 84.8 |  | 1 | 7 | 21.2 | 24.2 |
| 2 | 4 | 12.1 | 97.0 |  | 2 | 4 | 12.1 | 36.4 |
| 3 | 1 | 3.0 | 100.0 |  | 3 | 6 | 18.2 | 54.5 |
| 4 | 0 | 0 | 100.0 |  | 4 | 3 | 9.1 | 63.6 |
| 5 | 0 | 0 | 100.0 |  | 5 | 3 | 9.1 | 72.7 |
| 6 | 0 | 0 | 100.0 |  | 6 | 2 | 6.1 | 78.8 |
| 7 | 0 | 0 | 100.0 |  | 7 | 4 | 12.1 | 90.9 |
| 8 | 0 | 0 | 100.0 |  | 8 | 2 | 6.1 | 97.0 |
| 10 | 0 | 0 | 100.0 |  | 10 | 1 | 3.0 | 100.0 |
| Total | 33 | 100.0 |  |  | Total | 33 | 100.0 |  |

Note: *The number in Pathogens means the detected kinds of microbe species in sputum samples.

**Supplementary Table 2 The diagnostic criteria for pneumonia diagnosis in the study.**

| **Item** | **Manifestation** | |
| --- | --- | --- |
| 1 | Community morbidity. | |
| 2 | Pneumonia related clinical manifestations | |
|  |  | (1) Recent cough, sputum, or aggravation of existing respiratory symptoms, with or without purulent sputum, chest pain, dyspnea, and hemoptysis; |
|  |  | (2) fever; |
|  |  | (3) Signs of lung consolidation and/or smell and moist rales; |
|  |  | (4) Peripheral blood leukocytes >10×10e9/L or <4×10e9/L, with or without nuclear shift to the left. |
| 3 | Imaging examination of the chest revealed new patchy infiltrating shadows, leaf or segment consolidation shadows, ground glass shadows, or interstitial changes with or without pleural effusion. | |

Note: A clinical CAP diagnosis can be established by fitting 1, 3 and any item of 2, excluding pulmonary tuberculosis, pulmonary neoplasms, noninfectious pulmonary interstitial diseases, pulmonary edema, atelectasis, pulmonary embolism, pulmonary eosinophilic infiltration, and pulmonary vasculitis.
